# Subconjunctival injection of antagomir-21 alleviates corneal neovascularization in a mouse model of alkali-burned cornea

**DOI:** 10.18632/oncotarget.14370

**Published:** 2016-12-30

**Authors:** Yun Zhang, Ting Zhang, Xiaoyun Ma, and Jun Zou

**Affiliations:** ^1^ Department of Ophthalmology, Shanghai Tenth People's Hospital Affiliated to Tongji University, Shanghai, China; ^2^ Department of Plastic Surgery, Shanghai East Hospital, Tongji University School of Medicine, Shanghai, China; ^3^ Shanghai Sixth People's Hospital Affiliated to JiaoTong University, Shanghai, China; ^4^ Department of Ophthalmology, Guanghua Integrative Medicine Hospital, Shanghai, China

**Keywords:** microRNA sequencing, antagomir-21, alkali-burned cornea, corneal neovascularization, Sprouty

## Abstract

Corneal neovascularization may result in loss of corneal transparency and blindness. However, developing successful and inexpensive medical treatments for corneal neovascularization remains an unresolved issue. Recently, several studies have implicated miRNA functions in the regulation of cornea homeostasis. This study aimed to identify the miRNA expression profile in the neovascularized cornea after an alkali burn and to investigate the related underlying mechanisms. Here, alkali-burned corneas and matched normal tissues were pooled to perform miRNA sequencing. MiR-21 in alkali-burned cornea showed the greatest increment of abundance at 4 and 7 d after injury compared to the healthy cornea. The miR-21 expression was positively correlated with both the mRNA and protein level of key angiogenic factors including vascular endothelial growth factor (VEGF)-A and hypoxia-inducible factor-1α (HIF-1α). At 2 and 8 d after alkali burn, the mice received subconjunctival injections of antagomir-21 (1 or 5 nmol per injection). The injection of antagomir-21 (5 nmol) inactivated miR-21 and attenuated neovascularization progression by inhibiting the expression of VEGF-A and HIF-1α. Western blot analysis of the corneas demonstrated that antagomir-21 restored Sprouty 2/4 expression and silenced p-ERK activation. Therefore, these data reveal that antagomir-21 ameliorates the progression of corneal neovascularization likely via Sprouty 2/4-mediated inactivation of p-ERK. Delivery of antagomir-21 might be a potential therapeutic approach to prevent or treat visual loss caused by corneal neovascularization.

## INTRODUCTION

Alkali burn injuries to the eye frequently lead to corneal neovascularization resulting in loss of corneal transparency and blindness. The disequilibrium of angiogenic and anti-angiogenic stimuli is thought to be the underlying cause of corneal neovascularization. However, developing successful and inexpensive medical treatments for corneal neovascularization remains an unresolved issue.

MicroRNAs (miRNAs) are a class of non-coding RNAs, which are 22-nucleotide in length and can specifically bind to complementary sequences on the 3’UTR of target mRNAs and typically silence genes [1, 2]. Recently, several studies have implicated miRNA functions in the regulation of cornea homeostasis. MiRNAs including miR-205 [3, 4], miR-204 [5] and miR-424 [6] have been associated with corneal wound healing. Moreover, the cornea is an ideal organ for miRNA therapy due to its ease of access and angiogenic privilege. It has been reported that the treatment of diabetic organ-cultured corneas with miR-146a antagomir significantly enhanced cell migration and normalized epithelial wound healing [7]. MiRNAs regulate gene expression at the posttranscriptional level and play crucial roles in angiogenesis [8, 9]. As such, they may have a role in regulating corneal neovascularization. The identification of miRNAs as markers and modulators of corneal angiogenesis in various pathological conditions will provide us with gene-based therapies for corneal blindness.

In this study, Illumina miRNA sequencing technology was used to detect the miRNA expression profiles in the corneas after an alkali burn. The results showed that in injured tissues, seventeen miRNAs were upregulated and four miRNAs were downregulated, both by more than 2-fold. MiR-21a-5p (miR-21) showed a significant elevation in alkali-burned cornea and had a strong association with corneal neovascularization. Therefore, we attempted to inhibit the expression of miR-21 after alkali burn and observed the effect on corneal neovascularization progression and related angiogenic factors.

## RESULTS

### miR-21 is abundantly expressed and significantly upregulated during corneal neovascularization development in an alkali-burn model

The corneal neovascularization assay demonstrated that corneal neovascularization appeared at 4 d after alkali burn, progressed rapidly until 14 d, and regressed from 21 to 28 d, as observed using slit lamp microscopy (Supplementary Figure 1). The corneal neovascularization area reached a maximum at 14 d (Supplementary Figure 1). H&E staining showed that corneal neovascularization was most obvious at 14 d (Supplementary Figure 2). The number of corneal neo-vessels determined by CD31 staining remained high at 7 and 14 d and then returned to almost baseline by 28 d (Supplementary Figure 3).

**Figure 1:**
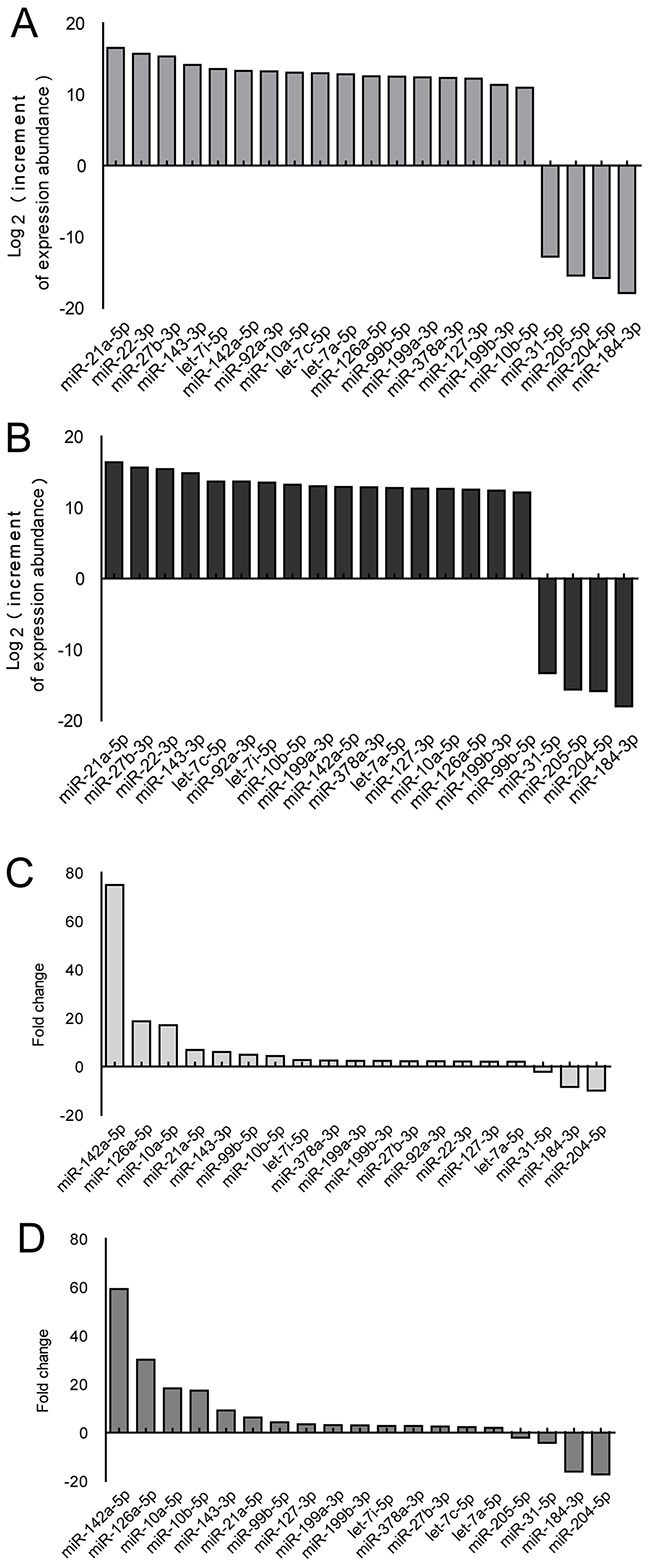
Screening of the miRNAs differentially expressed in corneas using the mouse alkali-burn model **A-D**. miRNA expression in whole corneas using the alkali-burned model and healthy controls was determined using Illumina miRNA sequencing analysis, and differential miRNAs (fold change>2) were sorted by increment of abundance and fold change. Injured cornea were harvested at 4 (A, C) and 7 d (B, D) after alkali burn, *n* = 3 per group.

Corneal tissue for miRNA sequencing was harvested at 4 and 7 d after alkali-burn injury, which is the early stage of neovascularization. MiR-21 showed the greatest increment of abundance at both time points (Figure 1A, 1B) and exhibited a significant fold change at these time points (Figure 1C, 1D). We arranged the order of miRNA by increase in abundance because both the abundance and the fold change of miRNA are important factors.

**Figure 2:**
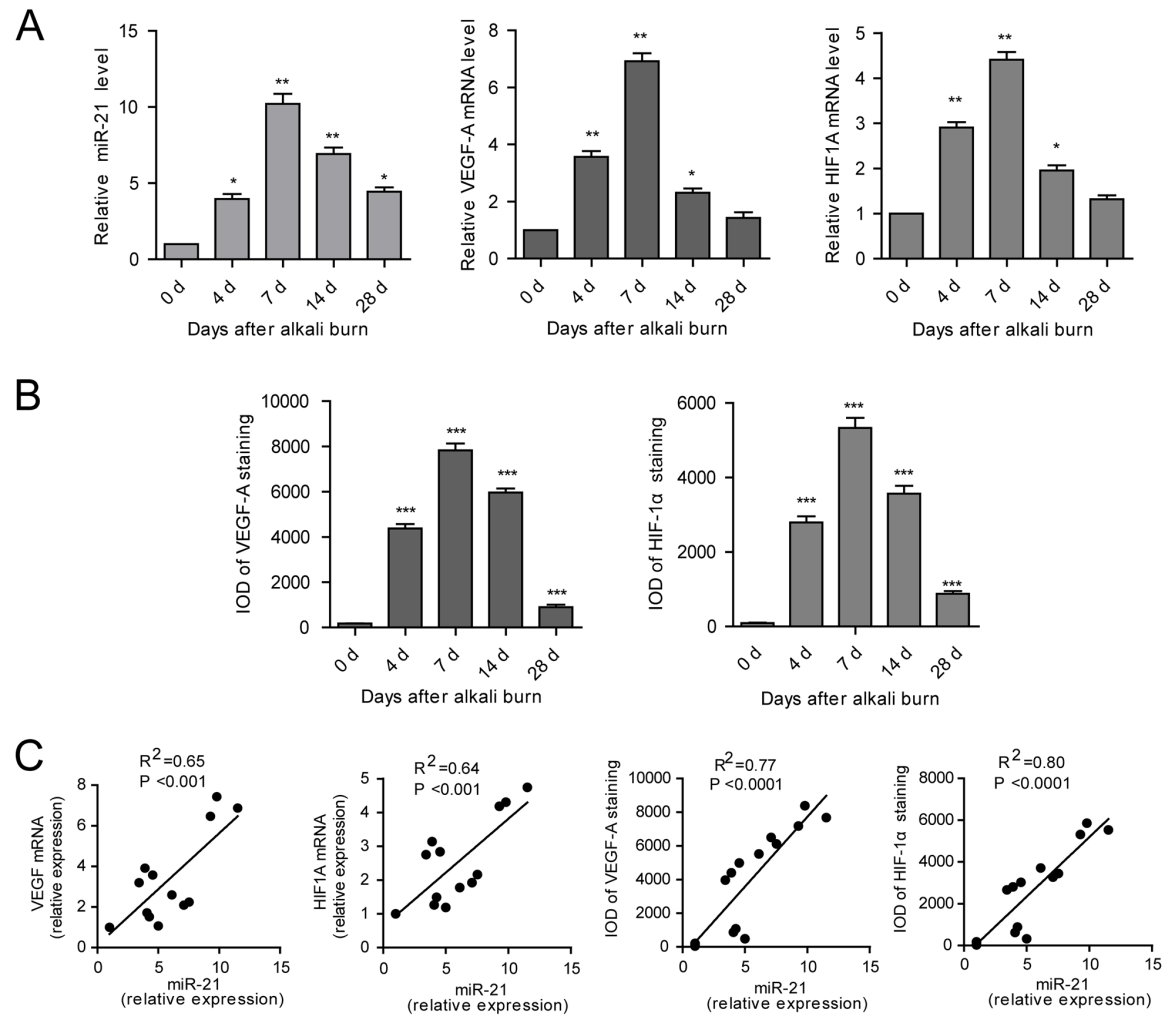
miR-21 expression is upregulated in alkali-burned corneas and correlated with the expression of VEGF-A and HIF-1α **A, B**. The qPCR analysis of miR-21, VEGF-A and HIF-1A (A) and immunohistochemical (IHC) IOD of VEGF-A and HIF-1α (B) in the cornea from the alkali-burned model at 4, 7, 14, 28 d after injury and the normal control. Data are represented as mean +/− SEM. *P < 0.05, **P < 0.01, ***P < 0.001 compared to day 0, *n* = 3 per group. **C**. Correlations between the expression of miR-21 and expression of VEGF-A and HIF-1α in cornea using the alkali-burned model and the normal control.

qPCR demonstrated that, after injury, miR-21 expression was greater at 4 d compared to day 0, remained high until 14 d and then returned to the level reached at 4 d by 28 d. As determined by qPCR, VEGF-A and HIF1A were significantly upregulated in the alkali-burned corneas from 4 to 14 d after injury and then returned to the approximate baseline level by 28 d (Figure 2A). Immunohistochemical staining further confirmed that expression of VEGF-A and HIF-1α was high from 4 to 14 d with a maximum at 7 d (Supplementary Figure 4, Figure 2B). The linear correlationanalyses showed that miR-21 expression strongly correlated with both the mRNA and protein level of VEGF-A and HIF-1α (Figure 2C).

### *In vivo* inhibition of miR-21 suppresses corneal neovascularization progression in the alkali-burned model

**Figure 3:**
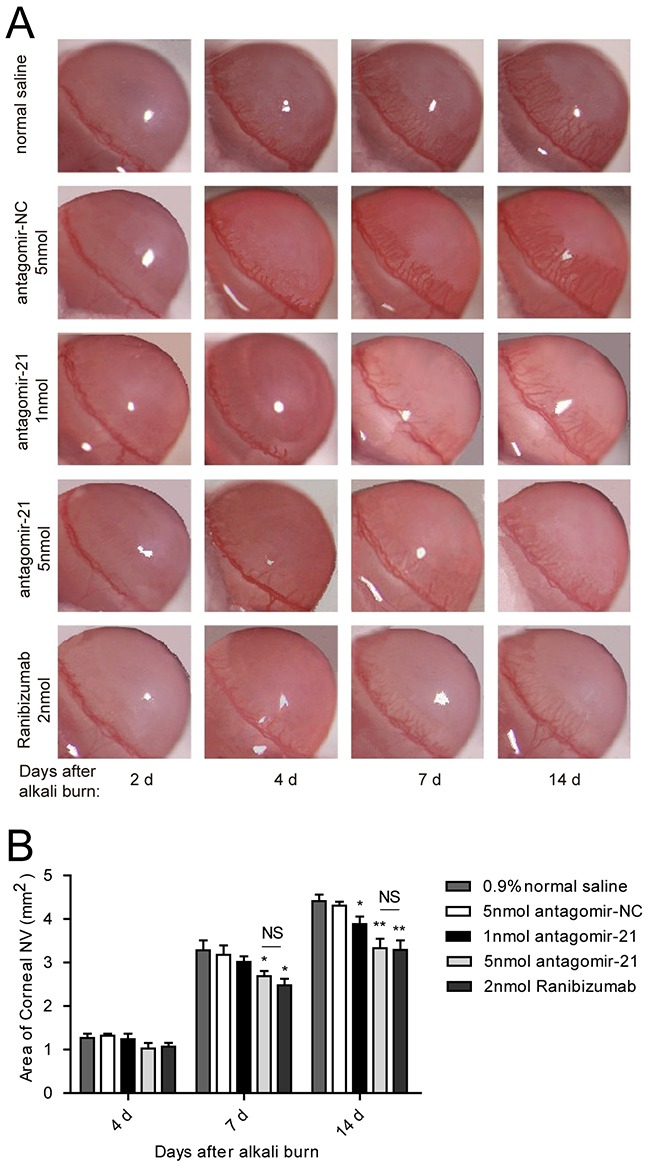
Effect of antagomir-21 on corneal neovascularization in the alkali-burned corneas **A**. Representative gross images of eyes treated with antagomir-21 (1 nmol or 5 nmol per injection), negative (normal saline and antagomir-NC) or positive (ranibizumab) controls at 2, 4, 7, 14 d after injury. **B**. Neovascularization area of the cornea. Data are represented as mean +/− SEM. *P < 0.05, **P < 0.01 compared to antagomir-NC. NS, no significant difference between the indicated groups, *n* = 8 per group.

**Figure 4:**
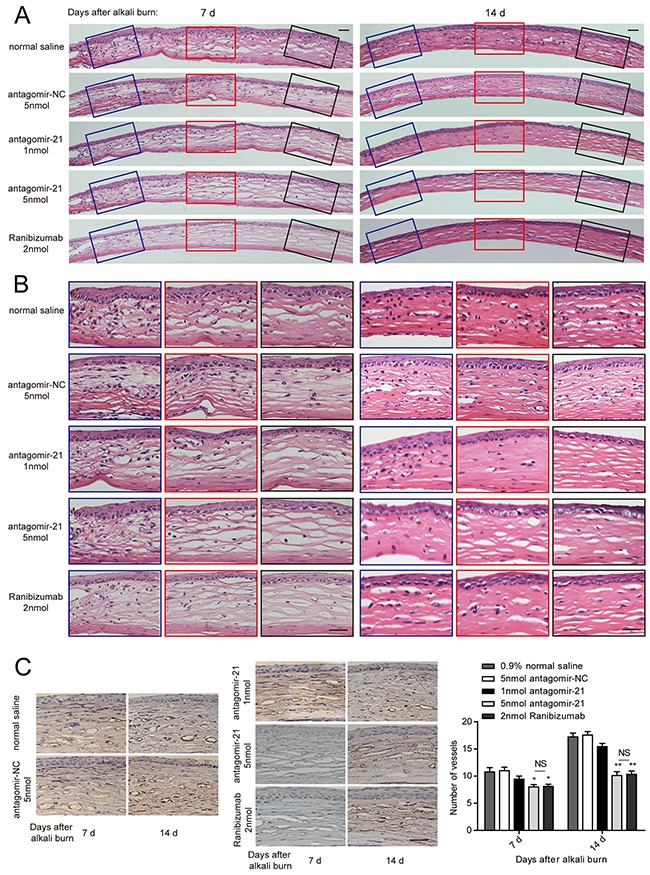
Histology of the cornea and the number of vessels revealed by CD31 immunostaining **A, B**. Representative H&E staining of entire cornea (A) and enlarged image of the left, middle and right parts (B) treated with antagomir-21, the negative or positive controls at 7 and 14 d after injury. Scale bar: 20μm. **C**. Representative CD31 staining image and vessel number counting in the alkali-burned cornea. Scale bar: 20μm. Data are represented as mean +/− SEM. *P < 0.05, **P < 0.01 compared to antagomir-NC. NS, no significant difference between the indicated groups, *n* = 6 per group.

To evaluate whether inhibiting miR-21 would prevent corneal neovascularization in an experimental animal model, we administered a subconjunctival injection of antagomir-21. Ranibizumab was used as a positive control, whereas the scrambled sequence (antagomir-NC) was used as a negative control. We would like to explore the therapeutic effect of antagomir-21 on early stage of corneal neovascularization. Therefore, the first injection time was selected at 2 d after injury. Antagomir-21 at a dose of 5 nmol inhibited neovascularization development (significant from 7 to 14 d; Figure 3, Figure 4) determined by the neovascularization assay, H&E staining and CD31 staining. Both the area and number of vessels treated by antagomir-21 (5 nmol) were not significantly different from that of ranibizumab. Treatment with 1 nmol of antagomir-21 showed a moderate decrease in the neovascularization area but not vessel number at 14 d (Figure 3, Figure 4).

### Antagomir-21 suppresses angiogenic factors

**Figure 5:**
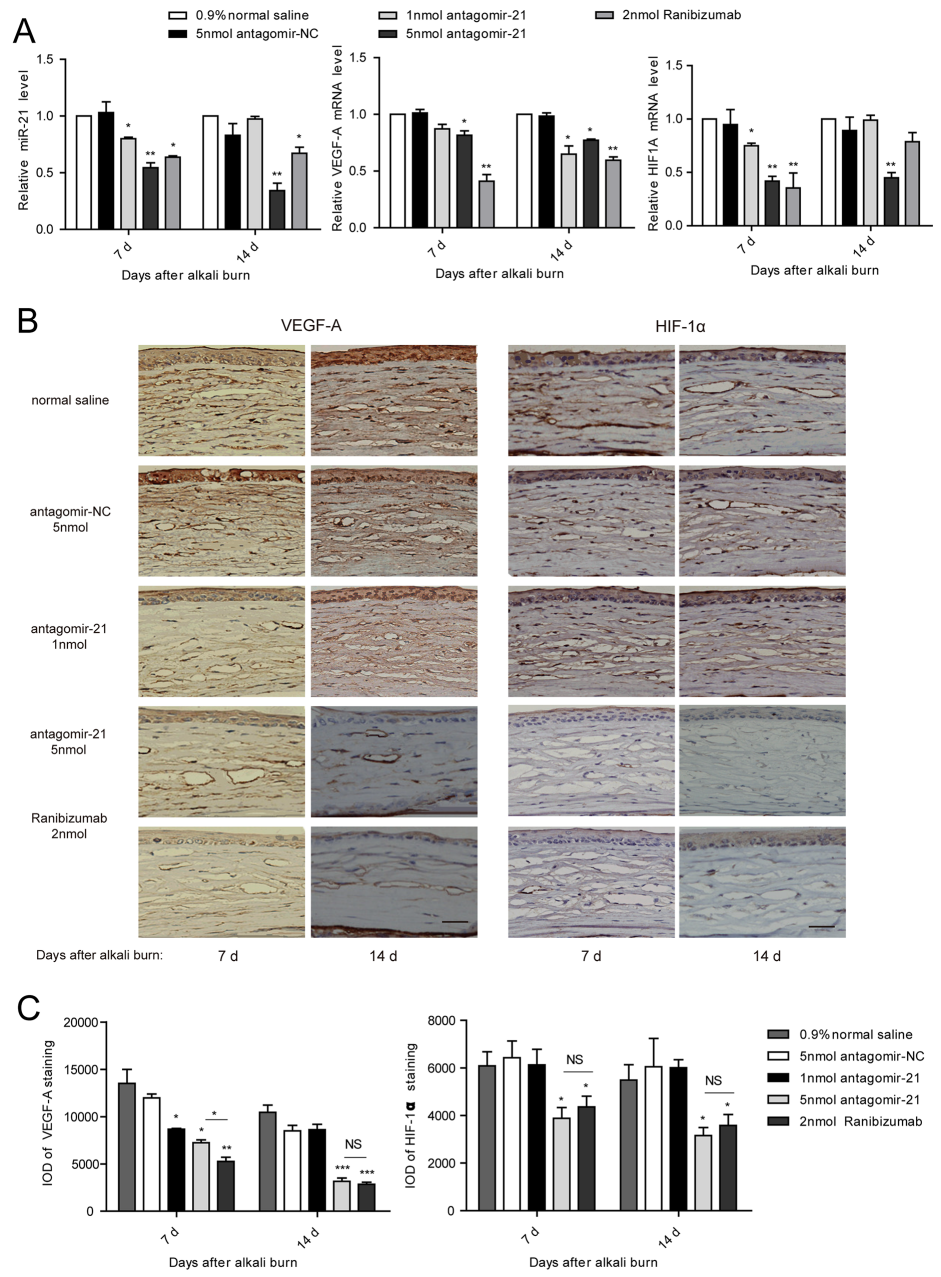
Antagomir-21 effectively suppresses VEGF and HIF-1 expression after corneal alkali burns **A-C**. qPCR analysis of miR-21, VEGF-A and HIF-1A (A), and IHC staining of VEGF-A and HIF-1α (B), and IOD of VEGF-A and HIF-1α (C) in the cornea from the alkali-burn model. Scale bar: 20μm. Data are represented as mean +/− SEM. *P < 0.05, **P < 0.01 compared to antagomir-NC. NS, no significant difference between the indicated groups, *n* = 3 per group in A and *n* = 6 per group in B.

Successful inhibition of miR-21 was confirmed by the qPCR measurement of the miR-21 level in the antagomir-21-injected corneas compared with antagomir-NC. However, ranibizumab also effectively suppressed the miR-21 level possibly as a result of neovascularization regression (Figure 5A). To elucidate the anti-angiogenic effect of miR-21, we detected the expression change of VEGF-A and HIF-1α. Through qPCR analysis, we observed that the level of VEGF-A was downregulated at 7 d and further decreased at 14 d due to the injection of 5 nmol antagomir-21. Moreover, 5 nmol antagomir-21 showed a potent inhibitory effect on HIF1A mRNA expression. Treatment with 1 nmol antagomir-21 demonstrated inhibition of HIF1A at 7 d only and inhibition of VEGF-A at 14 d only. Ranibizumab significantly decreased VEGF-A mRNA levels at 7 d and 14 d but inhibited the HIF1A level at 7 d only (Figure 5A). To confirm the gene expression results, we performed an immunohistochemical staining. As observed in Figure 5B, VGEF-A and HIF-1α was significantly decreased in the antagomir-21 (5 nmol) cornea compared with the normal saline group. This inhibition was comparable to the inhibition induced by ranibizumab (Figure 5C).

### The Sprouty (SPRY) /ERK axis is profoundly influenced by antagomir-21

**Figure 6:**
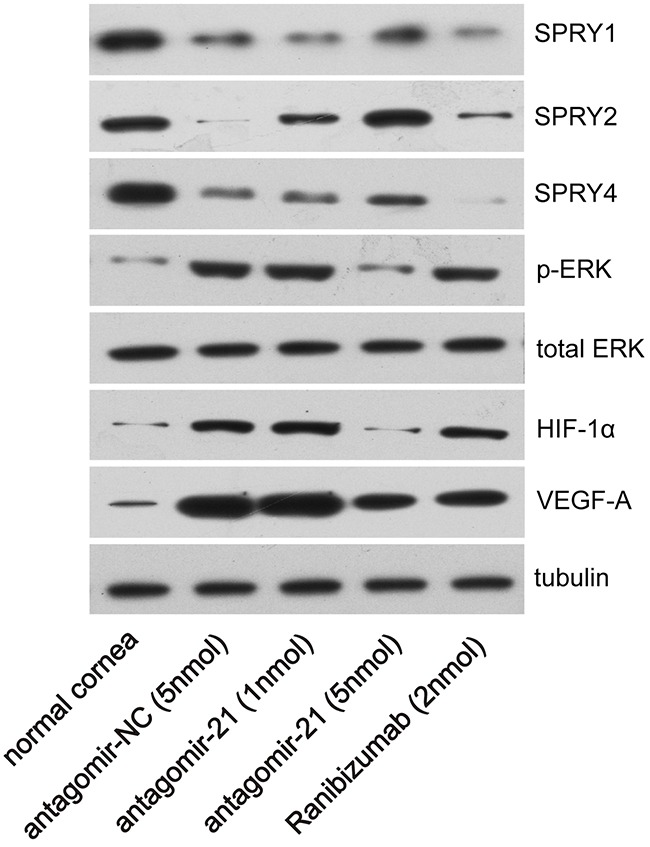
Effect of antagomir-21 on SPRY1/2/4, p-ERK, HIF-1α and VEGF-A expression after corneal alkali burns Western blot analysis of the indicated molecules in normal or alkali-burned cornea at 4 d after corneal alkali burns.

The SPRY family can be targeted by miR-21 in various cell or tissue types [10–13] and is a well-known negative regulator of p-ERK. For example, antagomir-21 normalizes changes in SPRY1 expression in cardiac fibroblasts [10]. MiR-21 represses SPRY2 expression during the adipogenesis and osteogenesis of mesenchymal stem cells [11]. We measured molecules of related signaling pathway at 4 d after injury since they are activated before angiogenesis. In our experiment, SPRY1/2/4 were significantly inhibited in the alkali-burned cornea at 4 d after injury compared with normal corneas (Figure 6). Therefore, we examined whether antagomir-21 recovers expression of the SPRY family in the alkali-burned cornea. We found that antagomir-21 stimulated SPRY2 at a low dose (1 nmol), but a greater level of SPRY2 and SPRY4 was achieved using a high dose of antagomir-21 (5 nmol). Specifically, SPRY2 was recovered to the normal level using 5 nmol antagomir-21. SPRY1 remained low after both a high or low dose of antagomir-21. Furthermore, antagomir-21 (5 nmol) completely inactivates p-ERK and abolished HIF-1α accumulation. VEGF-A expression after antagomir-21 (5 nmol) administration was reduced to the level observed after ranibizumab administration (Figure 6). In contrast, ranibizumab only induced a slight increase in SPRY2 and had no effect on p-ERK and HIF-1α expression at 4 d after injury (Figure 6).

## DISCUSSION

The normal cornea actively maintains avascularity, which is known as “angiogenic privilege”, and is required for corneal transparency [14]. Corneal neovascularization may occur in any of the layers of the cornea following ocular trauma, infection, etc. and leads to corneal opacity. An alkali-burned cornea is an outstanding model to observe the sprouting of blood vessels and to test antiangiogenic therapies. Here, we demonstrated that corneal neovascularization development induced by an alkali burn was accompanied by an elevation of miR-21 along with an increased expression of VEGF-A and HIF-1α. The *in vivo* administration of antagomir-21 greatly decreased the expression of VEGF-A and HIF-1α leading to a significant reduction in neovascularization progression. We speculate that the SPRY family are the possible targets of miR-21. Western blot analysis demonstrated that SPRY2 and SPRY4 were recovered, and p-ERK was suppressed in antagomir-21-treated corneas compared with the negative controls.

In 2015, An et al [5] reported the results of miRNA profiling during mouse corneal epithelial wound healing without neovascularization and performed an *in vitro* functional study of miRNA-204. In our study, we tried to modulate miRNA expression *in vivo* by subconjunctival injections, which would interfere with miRNA level in all three layers of cornea. Therefore, the whole cornea was used for miRNA sequencing rather than merely the corneal epithelium. Several differential miRNAs identified in our data, such as miR-21, miR-99b, miR-204 and miR-184, were also found in An's profiling data, implying their abundant expression in the corneal epithelium. MiR-143, -10b and -126, which are upregulated after an alkali burn, have been reported to be localized in the limbal epithelium [15]. In addition, miR-31, which was decreased in alkali-burned corneas, has been shown to promote corneal keratinocyte differentiation [16] and to inhibit the motility of human corneal keratinocytes [3]. Taking these reports together, our profiling indicates that miRNAs may be involved in complex pathological processes after alkali burn including corneal wound healing, limbal epithelium events and the differentiation and adhesion of keratinocytes. However, whether miRNAs are involved in corneal neovascularization remained largely unknown.

VEGF is considered to play a pivotal role in the proliferation and migration of vascular endothelial cells into the surrounding tissues [17, 18]. In alkali-burned corneas, the nanoparticle-mediated delivery of shRNA to VEGF-A inhibited neovascularization [19]. However, the mechanism of VEGF production has not been completely elucidated. In this alkali-burn model, we observed that the corneal neo-vessel number achieve a high level from 7 to 14 d and that the neovascularization area increased progressively until 14 d. In contrast, expression of miR-21 was the greatest at 7 d prior to neovascularization growth, implying the possibility that neovascularization may be enhanced by miR-21. Because miR-21 showed the greatest increment of abundance and was strongly correlated with angiogenic factors, we selected miR-21 for further experiments.

miR-21 was found to be upregulated in various solid tumors including breast cancer, colorectal cancer, gastric cancer, esophageal adenocarcinoma and glioblastoma [20–24], which implicates miR-21 as an ‘‘oncomir’’ in tumorigenesis. In recent years, the role of miR-21 in angiogenesis has received increasing attention. miR-21 is abundantly expressed in human endothelial cells [25]. Overexpression of miR-21 in DU145 cells enhanced HIF-1α and VEGF expression and induced tumor angiogenesis [26]. miR-21-deficient mice display a reduced number of infiltrated monocytes and impaired post-ischemic angiogenesis [27]. Moreover, Thum et al. showed that miR-21 is a mediator of ERK-MAPK signaling in cardiac fibroblasts [10]. In this study, the inhibitory effect of antagomir-21 on neovascularization was potent and comparable to ranibizumab. We observed a strong inhibition of HIF-1α at 7 d and VEGF-A at 14 d when using antagomir-21, implying that decrease of VEGF-A is possibly due to HIF-1α suppression by antagomir-21. SPRY2 and SPRY4 were recovered to a normal level through the use of antagomir-21, and p-ERK was significantly inhibited. The alteration of the SPRY/ERK axis is likely mediating the anti-angiogenic effect of antagomir-21.

In addition, other miRNAs detected in our profiling data need further investigation related to corneal neovascularization. For example, miR-27b was identified as a pro-angiogenic miRNA [25], and miR-378 is involved in tumor angiogenesis [28]. miR-99b is capable of potentiating the differentiation of human embryonic stem cells to vascular endothelial cells [29].

In summary, antagomir-21 suppressed corneal neovascularization development by reducing the expression of HIF-1α and VEGF-A. Our study provides evidence to support that miR-21 may be a useful target for corneal neovascularization management.

## MATERIALS AND METHODS

### Animals

Eight-week-old BALB/c mice (Shanghai Lab. Animal Center, China) were used. Mice were allowed to acclimatize for 1 week prior to experimentation. Postoperatively, all animals received a single 5 mg/kg dose of subcutaneous carprofen. Carbon dioxide inhalation and subsequent cervical dislocation were used to euthanize the animals. All experimental protocols were approved by the Animal Care and Use Committee of the Shanghai Tenth People's Hospital Affiliated to Tongji University in accordance with the ARVO Statement for the Use of Animals in Ophthalmic and Vision Research.

### Corneal alkali burn model

The mice were anesthetized using an intra-peritoneal injection of pentobarbital (40 mg/kg) supplemented with topical anesthesia (0.5% proparacaine hydrochloride, Alcon, USA). A corneal alkali burn was created in the right eye of the mouse by applying a paper disc (2.0-mm diameter) soaked with 2 μl of 0.5 M NaOH in the central cornea for 30 seconds aided by a surgical microscope. The ocular surface was then rinsed with 0.9% saline (10 mL) for 1 min.

### Animal treatment

Two days after the alkali injury, mice were randomly assigned to one of the five groups. Antagomirs (Ribobio technology, Guangzhou, China) were dissolved in normal saline. Ten microliters of antagomir-21 (5’-UCAACAUCAGUCUGAUAAGCUA-3’, 1 nmol or 5 nmol per injection), ranibizumab (2 nmol), normal saline or antagomir-NC (scrambled sequence, 5 nmol) was injected into the subconjunctival space at 2 and 8 d after injury. Antagomirs are cholesterylated anti-mRNA molecules that inhibit endogenous mature miRNAs.

### Corneal neovascularization assay

Each cornea was observed using a slit-lamp microscope, and photographs were taken using a digital camera at 4, 7, 14, 21, 28 d after the alkali burn. For antagomir experiments, photographs were taken at 2, 4, 7, 14 d after the alkali burn. Each photograph was analyzed at the same magnification using Image-Pro Plus 6.0 (IPP 6.0) software. Through the use of a reticule, the vessel length (*L*) from the limbus and the number of clock hours (C) of the cornea involved were measured, and the vascularized area = C/12×3.1416[*r*^2^-(*r*-*L*)^2^][30], where *r* = 1.5 mm and is the radius of the mouse cornea. Investigators who performed this assay were not blinded to treatment.

### H&E staining and immunostaining of corneal neovascularization

Corneas were carefully dissected under microscopy and rinsed in normal saline. Paraformaldehyde-fixed corneas were embedded in paraffin and sectioned at 4 μm for histological analysis. Each cornea yielded 12-16 slides for histological and immunohistological analysis. For the visualization of three layers of the cornea structure, three non-consecutive sections were stained with hematoxylin and eosin (H&E) in each cornea.

To observe the expression of angiogenic factors, sections were incubated with primary antibodies to mouse VEGF-A (1:150, Sangon Biotech, Shanghai, China) and HIF-1α (1:150, Sangon Biotech, Shanghai, China) at 4°C overnight. This was followed by three rinses in PBS. The staining was visualized through the use of a horseradish peroxidase (HRP) detection system (Dako), followed by counterstaining with hematoxylin. The integral optical density (IOD, IOD = average optic density × positive area) were quantified using Image-J software. We stained the corneas for marker of blood vessels (CD31, 1:100, Sangon Biotech, Shanghai, China). The number of vessels on microscopic images was quantified by two independent researchers, resulting in an inter-evaluator variation of less than 10%. Three non-consecutive sections were determined in each cornea and the researchers were not blinded to treatment.

### miRNA sequencing

We performed small RNA sample preparation according to the guide on the Illumina website. Briefly, total RNA was extracted using the mirVana^TM^ miRNA isolation kit (Austin TX, US) according to the manufacturer's instructions. RNA was electrophoresed using an Agilent 2100 Bioanalyzer (Agilent Technologies, Santa Clara, US), and all samples passed the quality test (RNA integrity number > 7.0, 28S/18S > 0.7). miRNA sequencing was performed using a Solexa GAII from Shanghai Bio-Chip. A standard Solexa TruSeq-Small RNA sequence protocol was used.

### Quantitative polymerase chain reaction (qPCR)

Each sample was pooled from four corneas and homogenized in 500 μl TRIzol reagent (Life Technologies). Total RNA was extracted according to the manufacturer's instructions. Then, 0.5 μg of the RNA was reverse-transcribed to cDNA using the PrimeScript RT reagent kit (Takara). A stem-loop primer (5’-GTCGTATCCAGTGCAGGGTCCGAGGTATTCGCACTGGATACGACTCAACA-3’) was used for miR-21 reverse-transcription. and a specific primer (5’-AACGCTTCACGAATTTGCGT-3’) was used for reverse-transcription of U6. QPCR was performed using the ABI 7500 Fast System (Applied Biosystems). Glyceraldehyde 3-phosphate dehydrogenase (GAPDH) or U6 was used for normalization. For qPCR, VEGF-A primers were the following: forward 5’-CCTTCGTCCTCTCCTTACCC-3’, reverse 5’-AAG CCACTCACACACACAGC-3’. HIF1A primers: forward 5’-ACTGCCACCACTGATGAATCAAAAACAG-3’, reverse 5’- TTCCATTTTTCGCTTCCTCTGA GCATTC-3’. GAPDH primers: forward 5’-GACTTCAA CAGCAACTCCCAC-3’, reverse 5’-TCCACCACCCTG TTGCTGTA-3’. miR-21 primers: forward 5’-CGGCGG TTAGCTTATCAGACTGA-3’, reverse 5’-CCAGTGCA GGGTCCGAGGTAT-3’. U6 primers: forward 5’-CTC GCTTCGGCAGCACA-3’, reverse 5’-AACGCTT CACGAATTTGCGT-3’.

### Western blot analysis

Each sample was pooled from five corneas. Briefly, excised corneas were homogenized in 100 μL of ice-cold buffer containing 50 mM Tris-HCl pH 7.5, 125 mM NaCl, 1% NP-40, 5.3 mM NaF, 1.5 mM sodium pyrophosphate, 1 mM orthovanadate, and 1 mg/ml of a protease inhibitor cocktail (Roche). Proteins (5 ng protein per sample) were electrophoresed on a 10% SDS-PAGE. Proteins were transferred onto nitrocellulose membranes (Bio-Rad, Hercules, CA, USA) and were blocked with a 5% BSA solution. The membranes were incubated overnight at 4 °C with antibodies to the following proteins: SPRY1 (1:1500), SPRY2 (1:1000), SPRY4 (1:1000), p-ERK (1:2000), total ERK (1:2000), VEGF-A (1:2000) and HIF-1α (1:500). Antibodies for SPRY1/2/4, p-ERK, total ERK and tubulin were purchased from Cell Signaling Technology (Beverly, MA, USA). VEGF-A and HIF-1α antibodies were from Abcam (Cambridge, UK).

### Statistical analysis

The results were analyzed statistically using a one-way analysis of variance and paired-sample t-tests. A value of *P* < 0.05 was considered statistically significant.

The funders had no role in study design, data collection, data analysis, data interpretation or writing of the manuscript.

## SUPPLEMENTARY MATERIALS FIGURES AND TABLES


