# Supplementary material for: Subconjunctival injection of antagomir-21 alleviates corneal neovascularization in a mouse model of alkali-burned cornea
Source: Oncotarget. 2016 Dec 30;8(7):11797–808. doi: 10.18632/oncotarget.14370 (PMC5355305; doi:10.18632/oncotarget.14370)
Supplement: Supplementary file 1 [file oncotarget-08-11797-s001.pdf]

Subconjunctival injection of antagomir-21 alleviates corneal neovascularization in a mouse model of alkali-burned cornea

SUPPLEMENTARY FIGURES

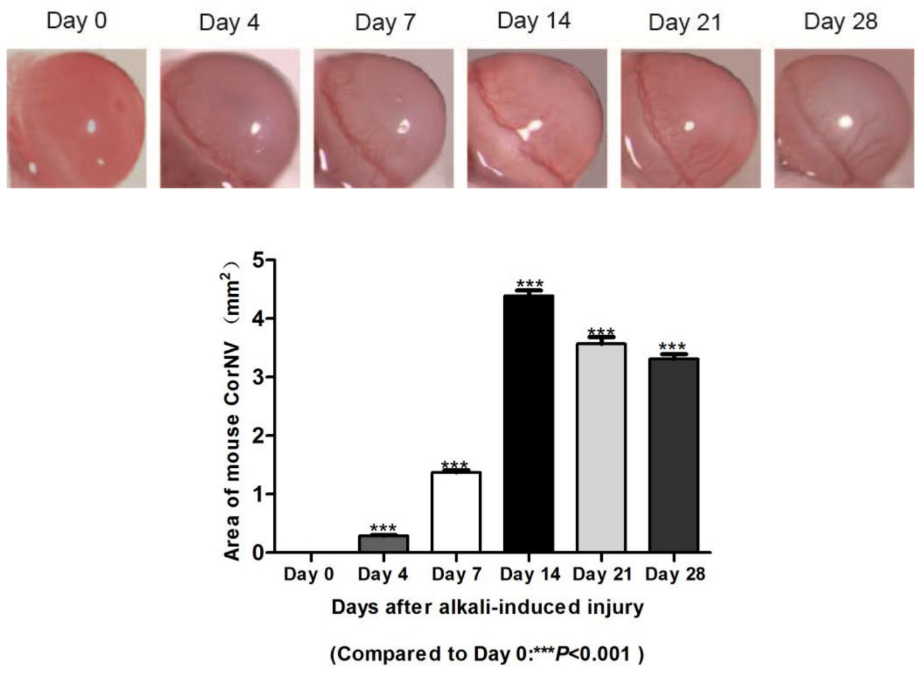

Supplementary Figure 1: Neovascularization development in the alkali-burned corneas. A. Representative gross view of eyes at 4, 7, 14, 21, 28 d after alkali burn and **B.** neovascularization area was determined. Data are represented as mean +/- SEM. ***P < 0.001 compared to day 0. n = 6 per group.

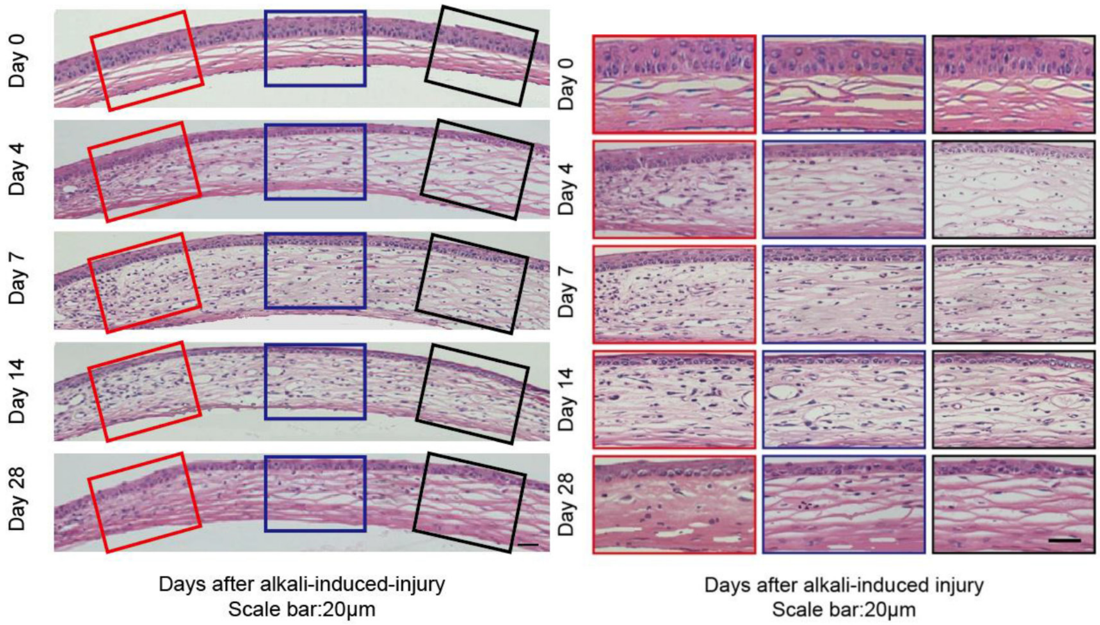

Supplementary Figure 2: Histology of the cornea after alkali burn. Representative H&E staining of entire cornea (left) and enlarged image (right) of the left, middle and right parts at 4,7,14 d and 28 d after injury. *n* = 3 per group

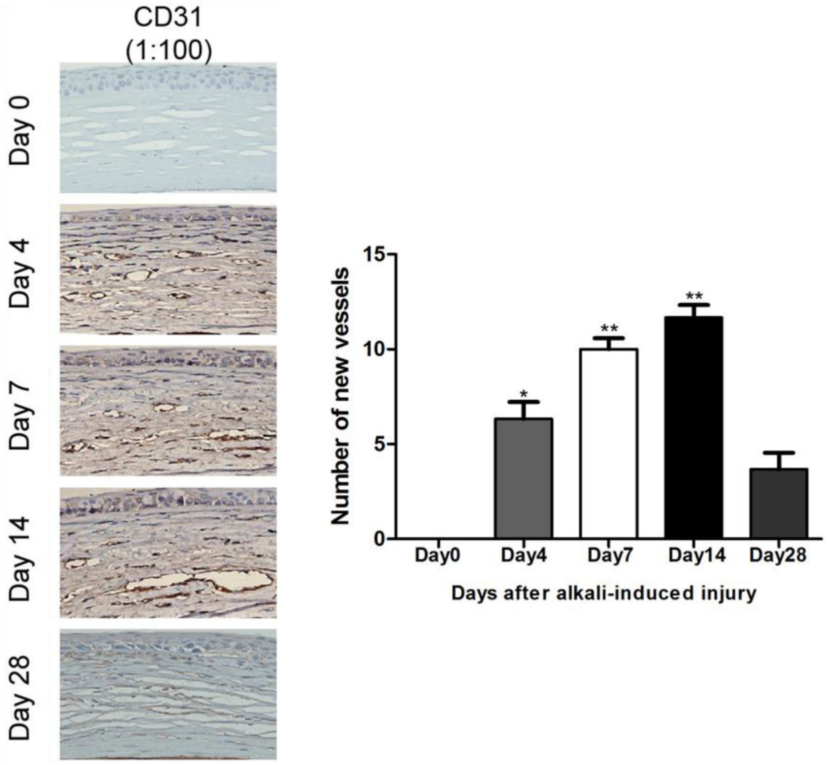

Supplementary Figure 3: The number of vessels in corneas revealed by CD31 immunostaining after alkali burn. Representative CD31 staining image and vessel number counting in the alkali-burned cornea. Data are represented as mean +/- SEM. $^{**}P < 0.01$ compared to day 0. *n* = 3 per group

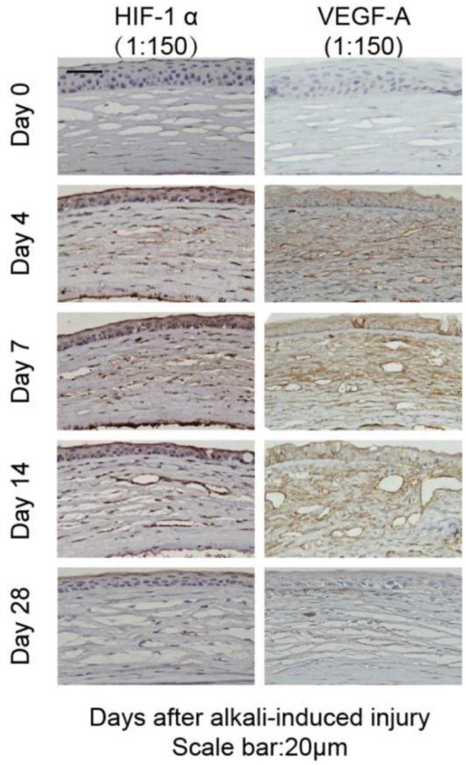

Supplementary Figure 4: Expression of VEGF and HIF-1α after corneal alkali burns. IHC staining of VEGF-A and HIF-1α in the cornea from the alkali-burn model. *n* = 3 per group
